# Endoscopic assessment of the J pouch in ulcerative colitis: A narrative review

**DOI:** 10.1002/deo2.373

**Published:** 2024-04-30

**Authors:** Shintaro Akiyama, Edward L Barnes, Tsubasa Onoda, Naoki Ishikawa, Mamiko Shiroyama, Yuka Ito, David T Rubin, Kiichiro Tsuchiya

**Affiliations:** ^1^ Department of Gastroenterology Institute of Medicine University of Tsukuba Tsukuba Ibaraki Japan; ^2^ Division of Gastroenterology and Hepatology University of North Carolina at Chapel Hill Chapel Hill USA; ^3^ Department of Gastroenterology NHO Mito Medical Center Ibaraki Japan; ^4^ Doctoral Program in Medical Sciences Graduate School of Comprehensive Human Sciences University of Tsukuba Tsukuba Ibaraki Japan; ^5^ University of Chicago Medicine Inflammatory Bowel Disease Center Chicago USA

**Keywords:** endoscopy, goblet cells, phenotype, pouchitis, ulcerative colitis

## Abstract

Patients with ulcerative colitis sometimes need a total colectomy with ileal pouch‐anal anastomosis due to medically refractory disease or colitis‐associated neoplasia. Up to 50% of patients with ulcerative colitis postoperatively develop pouchitis and the rate of chronic inflammatory pouch conditions requiring pouch excision or diverting ileostomy is reported to be 10%. In order to diagnose and monitor pouchitis, pouchoscopy is essential to assess endoscopic inflammatory findings of the J pouch and to survey neoplasia development, particularly in the remnant distal rectum. However, endoscopic protocols for the evaluation of the pouch may not be standardized worldwide and the reliability of existing disease activity indices for pouchitis has been questioned due to the lack of validation. Recently, reliable endoscopic scoring systems based on an observation of the anatomical location of the J pouch were reported and a significant association between the distribution pattern of endoscopic inflammation (i.e., endoscopic phenotype) and pouch outcomes was also uncovered. In this review, we discuss how to survey the J pouch using pouchoscopy, endoscopic indices for pouchitis disease activity, endoscopic phenotypes and classification, and the pathological mechanisms of pouchitis phenotype in patients with ulcerative colitis.

## INTRODUCTION

Patients diagnosed with ulcerative colitis (UC) require total colectomy with ileal pouch‐anal anastomosis in approximately 10% of cases due to medically refractory disease or colorectal neoplasia, with the ileal pouch‐anal anastomosis assuming the role of the rectum after surgery.^1^ However, up to 50% of patients develop acute pouchitis, and microbial alteration along with histologic colonic metaplasia of the ileal mucosa may be involved in the pathological mechanisms.^1^, ^2^, ^3^ In addition, 10%–15% of those who have acute pouchitis develop chronic pouchitis,^4^ requiring long‐term antibiotic therapies and biologics.^5^ Therefore, a certain number of patients suffer from pouch failure which requires diverting ileostomy or pouch excision due to stenosis or fistula formation.^6^ Since the incidence of pouchitis in UC has been increasing in recent years,^7^ there is a pressing need to understand appropriate strategies for pouch monitoring in the hopes of improving postoperative quality of life (QOL).

Pouchoscopy is useful to confirm endoscopic inflammation of the J pouch. The Pouchitis Disease Activity Index (PDAI) is the traditional assessment utilized in the management of pouchitis.^8^ However, existing instruments^8^, ^9^, ^10^, ^11^ to diagnose and monitor pouchitis have not been fully validated.^12^ A recent prospective study reported a novel reliable endoscopic scoring system for pouchitis, the Endoscopic Pouch Score (EPS), which was based on the segment approach utilized in the Simple Endoscopic Score for Crohn's Disease.^13^, ^14^ In addition, a distribution pattern of endoscopic inflammation (i.e., endoscopic phenotype) is considered an important factor contributing to pouch outcomes.^13^, ^15^ For instance, about 10% of patients with UC can develop Crohn's‐like disease of the pouch (e.g., a pouch phenotype with fistulas, stricture, or pre‐pouch ileitis) and this is the high‐risk phenotype for pouch failure.^4^, ^16^, ^17^ A distinct pouch phenotype, defined by chronic pouchitis with pre‐pouch ileitis, is more common in patients with UC and primary sclerosing cholangitis. This pouch phenotype is less responsive to standard antimicrobial therapy.^18^ A retrospective study conducted at the University of Chicago identified that endoscopic findings of pouchitis can be categorized into seven main phenotypes (i.e., the Chicago Classification of Pouchitis) and the “diffuse inflammation of the pouch body” phenotype significantly associated with the risk of pouch excision.^15^ These studies suggest that standardized endoscopic examinations according to an observation of each anatomical location of the J pouch may allow for the stratifying of patients based on the risk of pouch loss and can guide appropriate monitoring to improve pouch outcomes in patients with UC.^19^, ^20^

This review discusses endoscopic monitoring of the J pouch, indices for pouchitis disease activity, endoscopic phenotypes and their classification, and the pathological mechanisms of pouchitis in UC.

### Definition of pouchitis

Pouchitis is diagnosed based on an assessment of clinical symptoms, and endoscopic and histologic findings together. Sandborn et al. described the PDAI, as a composite score including clinical symptoms as well as endoscopic and histological scores.^8^ A subsequent study proposed that the modified PDAI (mPDAI), which excludes histological scores from PDAI, can provide a diagnostic accuracy that was comparable to PDAI for patients with acute pouchitis.^11^ Abdominal cramps, increased frequency of bowel movements, urgency, and pelvic discomfort are the most commonly reported symptoms of pouchitis.^11^ However, since these symptoms are not specific to pouchitis, the following differential diagnosis should be considered^21^: anal sphincter or pelvic floor dysfunction, decreased pouch compliance or emptying, pouch‐outlet obstruction, infections including cytomegalovirus and *Clostridioides difficile*, pouch or anastomotic stricture,^22^ cuffitis,^23^ irritable pouch syndrome,^24^ and small intestinal bacterial overgrowth.^23^, ^25^

Pouchitis is classically categorized into acute or chronic pouchitis.^26^ Acute pouchitis is defined as having symptoms lasting less than 4 weeks and responding to 2‐week courses of antibiotics. Chronic pouchitis is defined as having symptoms persisting more than 4 weeks despite standard antibiotics and requiring persistent use of antibiotics or anti‐inflammatory therapies.^27^ Based on the response to antibiotics, chronic pouchitis can be classified into antibiotic‐dependent and antibiotic‐refractory pouchitis.^26^, ^28^

The AGA Guideline on the Management of Pouchitis and Inflammatory Pouch Disorders has recently been published and offers practical definitions of pouchitis and other inflammatory pouch conditions.^29^ This guideline includes (1) intermittent pouchitis, (2) chronic antibiotic‐dependent pouchitis, (3) chronic antibiotic‐refractory pouchitis, and (4) Crohn's‐like disease of the pouch.^29^ Intermittent pouchitis is defined as isolated and infrequent episodes of classic symptoms of pouchitis which disappear with or without treatment, followed by prolonged periods of normal pouch function. Chronic antibiotic‐dependent pouchitis is defined as recurring episodes of pouchitis which respond to antibiotics, but relapse soon after ceasing them. To control symptoms, this type of pouchitis frequently requires continuous or recurrent antibiotics or other advanced therapies. Chronic antibiotic‐refractory pouchitis is defined as relapsing‐remitting or continuous pouchitis symptoms that inadequately respond to typical antibiotics, often requiring other advanced therapies. While the diagnostic criteria of Crohn's‐like disease of the pouch vary among literature, the definition of Crohn's‐like disease of the pouch in this guideline includes the presence of a perianal or another fistula that developed at least 12 months after the final stage of ileal pouch‐anal anastomosis surgery, the presence of prepouch ileitis, and stricture of the pouch body or prepouch ileum.^29^

### How to perform pouchoscopy for the J pouch in UC

**FIGURE 1:**
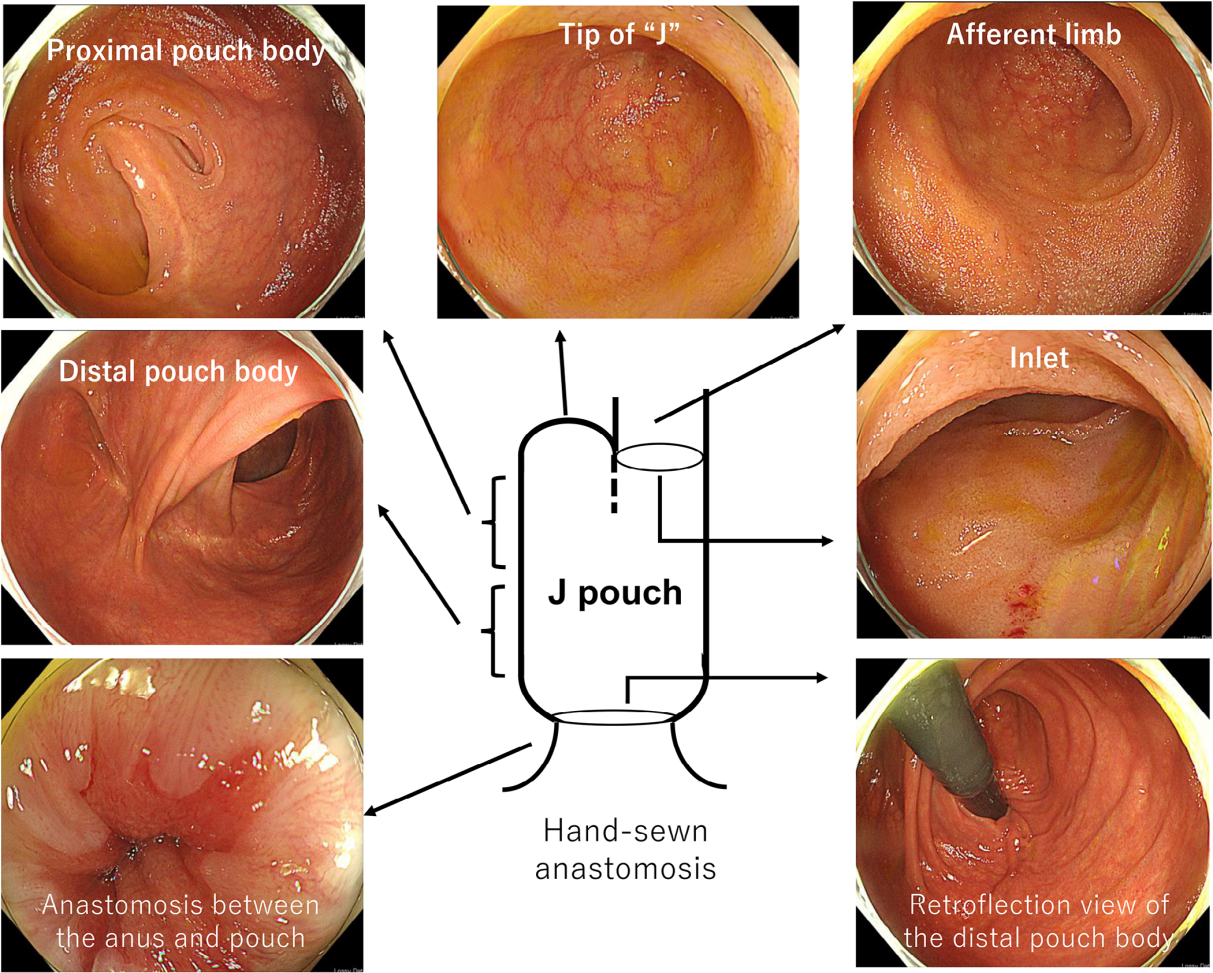
This patient had undergone total proctocolectomy with ileal pouch‐anal anastomosis. Since hand‐sewn anastomosis was performed, the rectal cuff was removed. Video S1 shows his examination. As only glycerin enema is used as a preparation, the mucosal surface should be cleaned using a water jet devised to obtain good visualization. It is better to choose an upper gastrointestinal endoscope or a thin lower gastrointestinal endoscope which facilitates retroflection in the distal pouch body. His pouchoscopy showed no inflammation at any anatomical location of the pouch. The endoscopic phenotype was normal according to the Chicago classification. The Endoscopic Pouch Score was 0. Endoscopic images of the anatomical location of the J pouch are extracted from Video S1.

**FIGURE 2:**
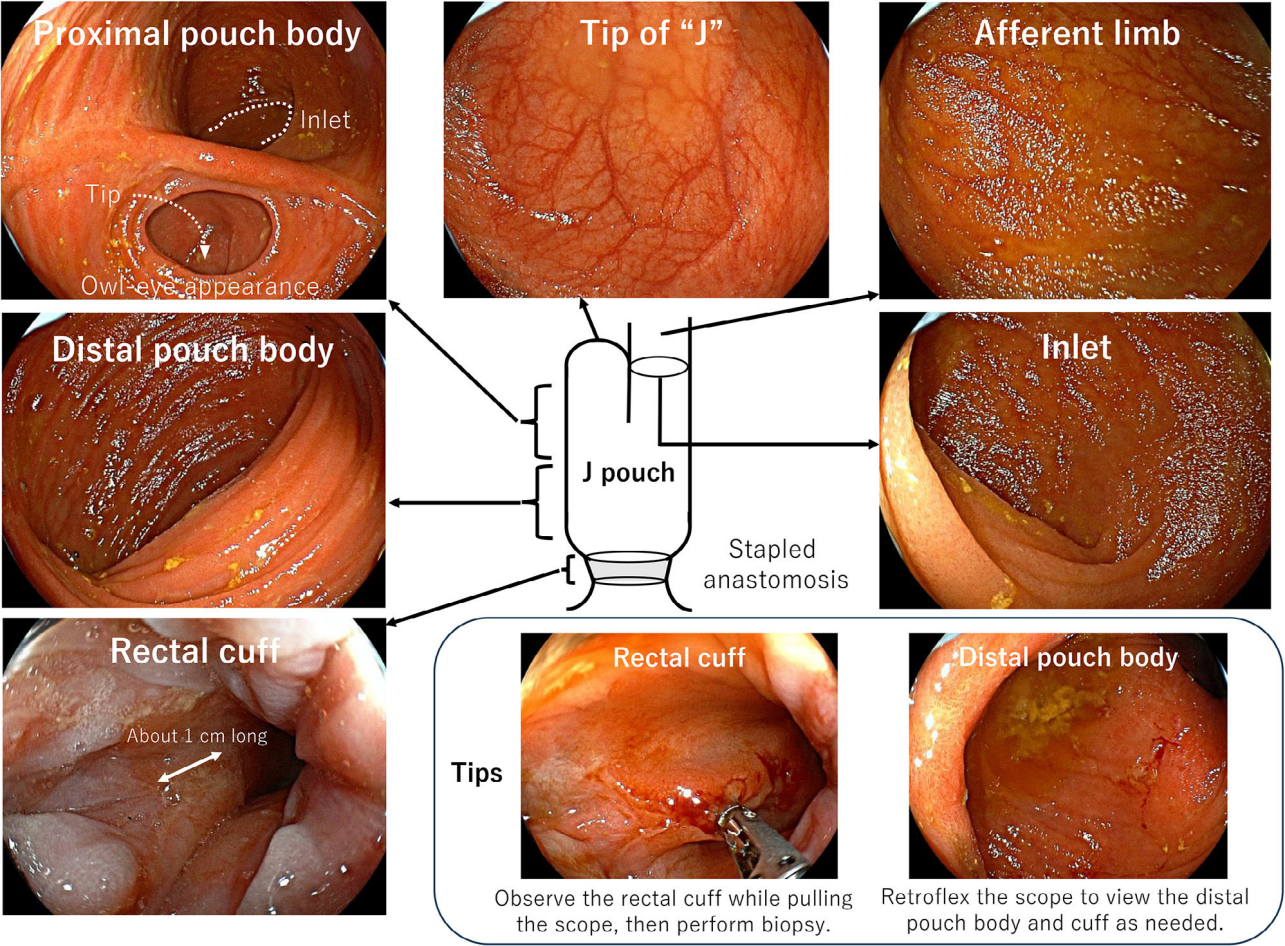
How to observe the J pouch in a patient with ulcerative colitis using pouchoscopy. This patient had undergone total proctocolectomy with ileal pouch‐anal anastomosis due to colorectal cancer. Since the stapled anastomosis was performed, the rectal cuff was still in place. Her pouchoscopy showed no inflammation at any anatomical location of the pouch. Biopsies were performed at the afferent limb, distal pouch body, and rectal cuff. The endoscopic phenotype was normal according to the Chicago classification. The Endoscopic Pouch Score was 0. Endoscopic images of the anatomical location of the J pouch are extracted from Video S2.

To evaluate the EPS and endoscopic pouch phenotype based on the Chicago Classification, endoscopists need to identify each anatomical location of the J pouch.^1^ Using pouchoscopy, the endoscopists record and report inflammatory findings at different anatomic areas of the J pouch: the afferent limb (i.e., the proximal pre‐pouch ileum), inlet (i.e., the distal pre‐pouch ileum), proximal and distal pouch, tip of the J, anastomosis, rectal cuff, anal canal, and perianal area (Videos S1 and S2 and Figures 1 and 2). Based on the PDAI, endoscopic inflammatory findings include ulceration, erosions/friability, erythema/edema, mucous exudates, loss of vascular pattern, stenosis, and granularity.^8^, ^11^ Since patients with stapled anastomosis have the rectal cuff (a remnant part of the distal rectum), an observation of the rectal cuff is essential to assess the disease activity of remnant UC (i.e., cuffitis) and to rule our neoplastic lesions. The length of the rectal cuff is usually about 1–2 cm, therefore it can be observed while pulling the endoscopy.^30^

According to the consensus guideline from the International Ileal Pouch Consortium, annual surveillance pouchoscopy is recommended for patients preoperatively diagnosed with colitis‐associated dysplasia or cancer.^31^ The underlying risk profile of patients determines the frequency of surveillance pouchoscopy. Regardless of preoperative diagnosis of colorectal neoplasia, surveillance pouchoscopy (every 1–3 years) is recommended for patients with other possible risk factors (i.e., primary sclerosing cholangitis, chronic pouchitis, Crohn's‐like disease of the pouch, long‐duration of UC, and family history of colorectal cancer in a first‐degree relative).^31^ For patients without these risk factors, surveillance pouchoscopy (every 3 years) is suggested.^31^ During the surveillance pouchoscopy, at least three biopsies are taken from the anal transition zone or cuff, along with biopsies from the afferent limb and pouch body (Video S2 and Figure 2). In addition, endoscopically evident lesions should be collected as well.^31^

### EPS and phenotype classification of pouchitis in UC

The most standard tool to diagnose and monitor pouchitis is PDAI or mPDAI as described above. Indeed, a recent double‐blind randomized trial assessing the efficacy and safety of vedolizumab for chronic pouchitis applied mPDAI‐defined remission, a reduction of ≥2 points from the baseline in the mPDAI total score, and an mPDAI score of ≤4.^32^ However, a systematic review indicated that PDAI may lack an acceptable level of interobserver reliability for some component items.^12^ A survey using modified RAND Appropriateness Methodology to assess the appropriateness of items in five endoscopic scoring systems^8^, ^33^, ^34^, ^35^, ^36^ showed that only endoscopic items of ulceration and ulcerated surface of the pouch body were found to have substantial reliability.^37^

A Prospective Registry for the Study of Outcomes and Predictors of Pouchitis and Pouch‐Related Disorders (the PROP‐RD Study) created the EPS which assessed inflammation not only inside but also outside the pouch body.^13^ Similar to the Simple Endoscopic Score for Crohn's Disease, the EPS employed segmental scoring.^14^ In this study, four experts reviewed 70 pouchoscopy videos in duplicate, in random order, and without clinical information, and the inter‐rater reliability of the EPS was calculated. The investigators found that the EPS demonstrated higher reproducibility among raters in both the overall scoring and many component items compared with the PDAI.^13^ Given that many patients with inflammatory conditions of the pouch can experience complications beyond the pouch body including strictures, cuffitis, and fistulae, the EPS attempted to account for these complications using modifier scoring as well. The EPS is a promising candidate for an objective and reliable measurement of pouchitis disease activity, including changes in disease activity over time.

On the other hand, PDAI and mPDAI only evaluate inflammatory findings of the pouch body alone and do not have items regarding endoscopic phenotype.^8^, ^11^ Endoscopic inflammatory status of the pouch has been categorized based on the location and distribution pattern: normal, afferent limb, inlet, focal inflammation of the pouch body, diffuse inflammation, cuffitis, and pouch with fistulas noted after 6 months from ileostomy closure.^15^, ^31^ This classification, so‐called the Chicago Classification of Pouchitis, was proposed by a recent retrospective study conducted at the University of Chicago that evaluated endoscopic findings at each anatomical location of the J pouch in patients with inflammatory bowel disease.^15^ This study revealed that each phenotype had different pouch outcomes, with the “diffuse inflammation of the pouch body” phenotype significantly associated with the risk of pouch excision.^38^ Their recent study also showed that endoscopic pouch phenotype and the risk of pouch loss can change over time. Subsequent development of diffuse inflammation, pouch‐related fistulas, and afferent limb or inlet stenoses significantly worsen the pouch outcomes, whereas pouch normalization was associated with favorable outcomes.^39^

Several subsequent studies conducted in Asian countries demonstrated that diffuse pouchitis based on the Chicago Classification exerted more serious impairment on the improvement of long‐term QOL.^38^ A retrospective study assessing our Japanese patients with UC unraveled that the order of endoscopic phenotypic frequencies was the same as for patients preoperatively diagnosed with UC in the original study of the Chicago Classification.^40^, ^41^ We also discovered that diffuse inflammation of the pouch body and pouch fistula were significantly correlated with the risks of diverting ileostomy as well as chronic pouchitis. Additionally, a recent study conducted in China revealed that patients with a combined phenotype of diffuse inflammation of the pouch body, inlet involvement, and cuffitis (i.e, the Diffuse‐Inlet‐Cuffitis phenotype) had poorer pouch function and QOL than those with other pouch phenotypes.^42^ All these findings imply that the Chicago Classification can be a universal one in defining endoscopic phenotypes and predicting pouch outcomes in UC.

All these studies highlight that the standardized and reliable endoscopic evaluation using pouchoscopy is important to stratify patients at higher risk of developing chronic pouchitis and pouch loss, and may provide an appropriate therapeutic approach to improve pouch‐related QOL in patients with UC.^19^, ^20^

### Contributing factors and pathological mechanisms of endoscopic phenotypes

While it is still largely unclear what risk factors or pathological mechanisms are involved in the development of pouchitis with poor outcomes, the original investigation of the Chicago Classification revealed that extensive colitis defined as the Montreal classification^43^ was significantly related to the development of diffuse inflammation of the pouch body, whereas extensive colitis was a negative predictor of the focal inflammation of the pouch body.^15^ This finding implicates that the preoperative disease extent of UC may determine endoscopic phenotypes. Further pathological analysis of colectomy specimens showed that patients with deep inflammation of the resected colon (e.g., deep fissuring ulceration, deep ulceration, knife‐like ulceration, and extensive ulceration with submucosal fibrosis) were at significantly increased risk of pouch fistula formation ≥6 months after ileotomy takedown. In addition, terminal ileal involvement, defined as any microscopic inflammation observed in the terminal ileum, was significantly associated with afferent limb involvement.^41^ This suggests that pathological findings of the resected colon can predict the subsequent endoscopic phenotypes of the J pouch and implicate similar pathogenetic mechanisms between the preoperative colonic inflammatory state and postoperative inflammatory pouch conditions.

Pouchitis in UC has also been reported to be linked to colonic metaplasia of intestinal goblet cells (GCs). Intestinal GCs secrete mucin and maintain a mucus gel layer that protects the surface epithelium.^44^ Intestinal GCs have functionally different subpopulations. For example, acidic mucin‐producing GCs are visible when intestinal tissues are stained with alcian blue. A distinct subpopulation of GCs produces sulfomucin, also known as colon‐type mucin, which is a form of acidic mucin that can be detected by high‐iron diamine (HID) staining. ^45^ While small intestinal GCs are negative for HID, colonic GCs in the human intestine are positive for HID.^46^, ^47^ There have been reports of ileal mucosal adaptations toward the colonic epithelium, particularly in UC patients suffering from pouchitis.^3^, ^48^, ^49^, ^50^ A comparative study evaluating differences between familial adenomatous polyposis and UC pouches revealed that sulfomucin expression was higher in mucous gel from the UC pouches. Additionally, this study demonstrated a correlation between higher expression of sulfomucin and increased colonization of sulfate‐reducing bacteria,^3^ implying that colonic metaplasia of GCs may alter the microbial profiles in the pouch and can induce inflammation.

In our study described above, we further examined colonic phenotypic changes in the ileal mucosa on pouch biopsy using HID staining according to the endoscopic phenotype of the Chicago Classification. As a result, the overall median percentage of sulfomucin‐producing colonic GCs on histological examination of 82 pouch biopsy specimens from 23 patients was 9.9%. The median rates of colonic GCs were 25.9% in diffuse inflammation without fistula, 10.3% in pouch fistula (± diffuse inflammation), and 6.0% in the other endoscopic phenotypes. Comparing biopsies from the other endoscopic phenotypes, the rate of colonic GCs was higher in biopsies from patients with diffuse inflammation or pouch fistula, suggesting that the proportion of colonic GCs is greater in pouch biopsies obtained from UC patients with poor endoscopic phenotypes. Our result suggests that colonic GC metaplasia may lead colonization of sulfate‐reducing bacteria (e.g., *Desulfovibrio* spp.)^46^ with hydrogen sulfide production, which induces epithelial apoptosis as well as mucosal depletion.^3^, ^51^ This mechanism may contribute to the diffuse inflammatory changes in the pouch body which lead to chronic pouch inflammation and deterioration of pouch‐related QOLs. We believe that HID staining of biopsy specimens may enable early detection and antibiotic therapy for endoscopic pouch phenotypes with poor outcomes in patients with UC.

## SUMMARY AND FUTURE DIRECTIONS

In this review, we introduced how to observe the J pouch and its timing for patients with UC and discussed current topics regarding endoscopic pouch assessment.

 Regarding future clinical research relevance, while many instruments to evaluate endoscopic pouch findings have been previously reported, few scoring systems are well validated. To establish reliable and objective endoscopic measures for the J pouch, multicenter prospective validation studies are needed. As for endoscopic phenotypes, diffuse pouchitis is defined as two or more endoscopic inflammatory findings in all anatomical locations of the pouch body (e.g., the tip of J, proximal, and distal pouch body) according to the Chicago Classification.^15^ Although other studies validated that diffuse pouchitis was significantly associated with poor pouch outcomes,^38^, ^40^ a simpler definition of this phenotype should be considered in actual clinical settings.

In terms of basic research relevance, given bacterial alteration corresponding to colonic intestinal metaplasia is implicated in the pathogenesis of endoscopic diffuse pouchitis,^40^ further investigations are warranted to analyze muti‐omics as well as multi‐biome and their trans‐kingdom interactions^52^ based on the endoscopic phenotypes. Organoid research is also helpful in knowing how gene expression patterns change in colonic GCs which emerge in the ileal mucosa of the pouch.

Further investigations focusing on the reliable measure for endoscopic assessment of pouchitis and its pathological mechanism are helpful to improve patients’ QOL and pouch outcomes in patients with UC.

## CONFLICT OF INTEREST STATEMENT

Tsubasa Onoda, Naoki Ishikawa, Mamiko Shiroyama, and Yuka Ito have no relevant disclosures. Shintaro Akiyama has received honoraria (lecture fees) from AbbVie and EA Pharma. Edward L Barnes has served as a consultant for TARGET RWE. David T Rubin has received grant support from Takeda; and has served as a consultant for Abbvie, Abgenomics, Arena Pharmaceuticals, Bellatrix Pharmaceuticals, Boehringer Ingelheim Ltd., Bristol‐Myers Squibb, Celgene, Syneos, Dizal Pharmaceuticals, Genentech/Roche, Gilead Sciences, Ichnos Sciences S.A., InDex Pharmaceuticals, Iterative Scopes, Janssen Pharmaceuticals, Lilly, Pfizer, Prometheus Laboratories, Reistone, Takeda, and Techlab Inc. Kiichiro Tsuchiya has received honoraria (lecture fees) from Takeda, AbbVie, and Janssen, and research grants from AIMO Pharmaceutical.

## Supporting information


**VIDEO S1** How to observe the J pouch in a patient with ulcerative colitis using pouchoscopy. This J pouch had a hand‐sewn anastomosis.
**VIDEO S2** How to observe the J pouch in a patient with ulcerative colitis using pouchoscopy. This J pouch had a stapled anastomosis.
