# Supplementary material for: Endoscopic assessment of the J pouch in ulcerative colitis: A narrative review
Source: DEN Open. 2024 Apr 30;5(1):e373. doi: 10.1002/deo2.373 (PMC11058686; doi:10.1002/deo2.373)
Supplement: Supplementary file 1 — VIDEO S1 How to observe the J pouch in a patient with ulcerative colitis using pouchoscopy. This J pouch had a hand‐sewn anastomosis. VIDEO S2 How to observe the J pouch in a patient with ulcerative colitis using pouchoscopy. This J pouch had a stapled anastomosis. [file DEO2-5-e373-s001.docx]

**Video links:**

[Video 1](https://wiley-my.sharepoint.com/:v:/p/yikegami/EdLuHE8PBtZJtq4HbXCjPvAB-7A1BCffVFOi4woyuSrvxw?e=p3YGbW)

[Video 2](https://wiley-my.sharepoint.com/:v:/p/yikegami/Edv2Hw5gcsRIrmagSH0EgtcBCwayNhGVWUQK2tg9dX1uGw?e=OtNCKh)
